# *Coalicion de Salud Comunitaria (COSACO)*: using a Healthy Community Partnership framework to integrate short-term global health experiences into broader community development

**DOI:** 10.1186/s12992-016-0155-y

**Published:** 2016-05-02

**Authors:** Lawrence C. Loh, Olga Valdman, Matthew M. Dacso

**Affiliations:** Dalla Lana School of Public Health, University of Toronto, 155 College Street, Sixth Floor, Toronto, ON M5T 3 M7 Canada; The 53rd Week, 151 Kent Avenue, Unit 116, Brooklyn, NY 11249 USA; Center for Global Health Education, University of Texas Medical Branch-Galveston, 301 University Blvd, Galveston, TX 77555 USA; University of Massachusetts Medical School, 373 Plantation Street #318, Worcester, MA 01605 USA

**Keywords:** Community health services, Collaboration, Partnership, Global health education, Development, Community health partnership, Community engagement in health

## Abstract

**Background:**

There is growing concern that short-term experiences in global health experiences (STEGH), undertaken by healthcare providers, trainees, and volunteers from high income countries in lower and middle income countries, risk harming the community by creating a parallel system of care separate from established community development efforts. At the same time, the inclusion of non-traditional actors in health planning has been the basis of the development of many Healthy Community Partnerships (HCP) being rolled out in Canada and the United States. These partnerships aim to bring all stakeholders with a role to play in health to the table to align efforts, goals and programs towards broad community health goals.

**Results:**

This methodology paper reports on the process used in La Romana, Dominican Republic, in applying a modified HCP framework. This project succeeded at bringing visiting STEGH organizations into a coalition with key community partners and supported attempts to embed the work of STEGH within longer-term, established development plans.

**Conclusions:**

In presenting the work and process and lessons learned, the hope is that other communities that encounter significant investment from STEGH groups, and will gain the same benefits that were seen in La Romana with regards to improved information exchange, increased cross-communication between silos, and the integration of STEGH into the work of community partners.

## Background

Recent increases in short-term experiences in global health (STEGH) participation has occurred both within and outside of broader development efforts [1]. The rising popularity of STEGHs has been driven by a diverse group of stakeholders, which broadly includes academic institutions sending learners on study abroad/alternative spring break programs, faith-based and community groups conducting charity work (e.g. medical clinics, service, or research, among others), and non-profit or for-profit organizations providing “voluntourism” experiences [2, 3]. Communities worldwide subsequently experience growing impacts resulting from hosting ever growing numbers of STEGH groups, and increasing controversy debates regarding the merits, impacts, and benefits of STEGH efforts [4].

Another contemporary trend has been the establishment of Healthy Community Partnerships (HCPs) in many jurisdictions. These are community coalitions that connect traditional health and healthcare stakeholders with non-traditional partners that influence community health, such as government, private sector (e.g. chamber of commerce), and community services (schools, police, and others) [5]. Developed with the recognition that many of today’s chronic diseases are influenced by social determinants of health, HCPs address community contexts that influence health by aligning the activities of constituent stakeholders towards addressing identified health priorities.

To address some of the controversy around STEGH, a novel process linking these two concepts was used to create a modified HCP framework in La Romana, Dominican Republic, entitled the *Coalicion de Salud Comunitaria* or COSACO. The key modification to the COSACO HCP framework was the inclusion of visiting STEGH groups, to integrate STEGH into broader community planning with the hopes of minimizing potential harms STEGH might have on the community. The wider goal imagined STEGH groups operating not in isolation or in parallel, but rather, within the local health system together with guidance and direction from key local stakeholders, especially local government and health authorities.

This review describes the process around the development of COSACO from initial problem formulation and the formation of a steering committee, followed by identification and invitation of stakeholders, and the timeline of work leading up to an inaugural HCP meeting attended by key stakeholders. Lessons learned in this process will help other communities use HCPs to better incorporate STEGH teams into established community plans for health and development.

## Methods

### Growing trends: Healthy community partnerships and short-term experiences in global health

First developed in Canada in the late 1980s and formalized at the international level by the World Health Organization’s “Healthy City” project in 1986, HCPs have broadly achieved results in various community settings. At the heart of this framework is a priority on capacity building and empowering individuals, organizations, and communities to contribute to community health [6, 7]. Reviews have demonstrated that the deployment of HCPs is a promising strategy for engaging community stakeholders and citizens in common efforts towards addressing the social determinants of health, and today, HCPs (and equivalent coalitions) can be found in many jurisdictions in high-income countries, notably in Canada, the United States, and Western Europe [8].

Over the same time period, the growing popularity of STEGH, particularly among health care trainees and young providers, has raised questions around disparities in benefits and harms assumed by different stakeholders. Literature documents clear benefits for visiting participants and their sending institutions, but for host communities, STEGH benefits remain less clear. These communities also bear a notable risk of harm arising from poorly conducted STEGH. At best, visitors represent an opportunity cost that consumes scarce local resources in providing culturally incongruent programs, but at worst, they may create parallel systems operating independent of local context, needs, or programming, resulting in competition for scarce resources and giving rise to conflict, redundancy and unsustainability [9].

Literature has increasingly attempted to describe factors that improve the conduct of STEGH to mitigate the potential risks on receiving communities. Principles include greater reciprocity, appropriate selection and preparation of volunteers, and a focus on visitor learning, humility and cultural sensitivity [3, 10]. Literature also calls on STEGH to be embedded within longer-term, locally-led development plans, as opposed to being conducted as stand-alone endeavours. In many cases, the harms arising from STEGH are driven in part by a lack of visitor interaction with local leadership and the perpetuation of power differentials and perceived stereotypes associated with visiting volunteers.

Improved partnership and collaboration thus represent a potential strategy to better integrate STEGH into the local developmental context, encouraging resource and data pooling among stakeholders, improved continuity, and joint project planning around locally described priorities [11, 12]. As a model for partnership, this project selected an HCP framework as a model that could draw key stakeholders together, bringing STEGH groups in with local government, local health and healthcare leadership, and other community stakeholders.

The applied HCP model aimed to minimize harms and optimize community outcomes related to STEGH in two ways: at minimum, an HCP could encourage communication between local stakeholders and STEGH groups, allowing them to operate with a basic understanding of local context and priorities. The broader hope, however, was that an HCP would align the activities of various STEGH with specific local efforts, breaking down silos between stakeholders towards incorporating STEGH into broader community health plans arising from the local community health ecosystem.

### A HCP in the Dominican: La Romana

La Romana is the third-largest community in the Dominican Republic, with a population of 130,000 in a census conducted in 2010. With sugarcane as a significant economic driver, the city is home to numerous *bateyes*, which are rural settlements traditionally inhabited by Haitian migrant workers employed by the sugarcane industry, though in recent times other marginalized groups call these settlements home.

Decades of systematic discrimination has led to the disenfranchisement of the *batey* population, limiting the access of these residents to health and social services. Since the mid-1980s, a number of faith-based groups in the United States and Canada have been engaged with a local partner to provide various charity services in the community, notably around healthcare, construction, and education. The number of health-focused visiting groups has continued to grow in both number and diversity, and today, faith-based groups have been joined by groups from academic institutions, non-profit organizations, and community service organizations.

The present context in La Romana involves a large mass of groups from abroad conducting health-related STEGH in the bateyes, usually operating with the assistance of a number of local partners. In discussions on the ground, described further in this paper, various groups identified concerns around redundancy and waste between STEGH, which in turn limited their overall impact on community health. Initial interest in collaboration arose from a small group of visiting organizations operating through a local mission hospital; discussions focused on the development of a partnership model to address identified issues.

### Building the initial collaboration

The development of COSACO was preceded by the development of a collaborative model between the local mission hospital and associated STEGH groups in 2008. Initial partners were a North American based non-profit organization, whose strategic goal at the time was to foster collaboration between groups, and a U.S.-based academic institution. An initial meeting arose from a request made to the local partner around contact information for other STEGH groups that volunteered in the community.

Initial discussions focused on improving continuity of care between 10-day long mobile medical clinic experiences conducted by visiting STEGH groups in partnership with the mission hospital. Targeted for inclusion were dozens of visiting STEGH groups with different skillsets, values, and goals, with the common thread being their partnership with the mission hospital. Over the next 4 years, the initial members of this collaborative partnership launched two different web platforms to encourage information sharing, with varying degrees of success, and also hosted annual symposia which brought together representatives from the local hospital and any and all STEGH organizations that worked with this partner in La Romana, to foster greater information sharing.

By 2012, this collaboration enabled participant groups to pool clinical encounter data from mobile medical clinics to inform a chart review of basic epidemiological trends observed in the work of STEGH medical clinics. The annual symposia grew to include presentations and discussions on standards, guidelines, and joint projects to be undertaken collectively. Growing interest in research and evaluation allowed many of these groups to jointly bring their results to major global health conferences, which in turn provided further opportunities to reach out to other potential collaborators working in La Romana.

### Broadening the net: towards COSACO

During the development of the initial collaboration, networking efforts quickly revealed that other local partners hosted STEGH groups in La Romana. Outreach at various global health conference identified a diversity of visiting STEGH groups that worked with different local partners in the La Romana area on various health-related topics ranging from direct service provision and health research to health policy and advocacy. A common thread was that many of these groups undertook their work independent of formal connections to local government or public health.

With this growing awareness, members from the initial collaboration reached out to new contacts from conferences in mid-2014 and identified a shared desire for a central data repository to help address various issues with the impact of STEGH within the La Romana community. Specifically, groups expressed concerns around a lack of clarity as to how or even whether STEGH fit into local health and development plans. Similar to the impetus for the first collaboration around the mission hospital, concerns were also expressed around the actual community impacts of STEGH work, redundancy and duplication around research and programming.

As a result of these discussions, summer 2014 saw the initial steps towards the development of COSACO which aimed to employ a HCP framework to better represent the view of local community leaders and stakeholders. A steering committee was formed, which consisted of 1–2 representatives from the leadership structures of six North American institutions and non-profits that worked in Romana and the three specific local non-profit partners and the local mission hospital they worked in partnership with. Selected by consensus, the chair of this process represented one of the U.S.-based non-profits, and representatives from two of the local non-profits agreed to provide on-the-ground logistic support. Initial discussions focused on the format of an HCP for La Romana and an assessment of the feasibility of implementation. This required identification of other stakeholders of interest that would be invited to join, discussions on potential manifestations for the HCP (e.g. a locally-held meeting), and identifying potential funding sources to support the process.

The steering committee agreed on launching the HCP at a major symposium held locally within the next year (before fall 2015), which would involve key players described in Table 1 to participate. Local input was crucially sought around the feasibility and interest of deploying an HCP model in La Romana, with aims to ensure that the concept could be communicated as easily as possible to invited stakeholders.

**Table 1:** Invited and participant stakeholders of COSACO

Health and healthcare (hospitals, clinics)
- Local public hospitals^a^
- Local mission hospital^a^
- Hospitals from neighbouring suburbs/exurbs of La Romana^b^
- Local private hospitals^a^
Government
- Provincial governor’s office^b^
- Ministry of public health^b^
- Public health department^b^
- Local city government
Non-profit sector
- Issue-focused non-profit organizations^a^
- Locale-focused non-profit organizations^a^
- Local academic institutions^a^
Private sector
- Key local employers/industry partners
Other community groups
- School district
- Law enforcement
- Immigration bureau
All visiting STEGH groups from U.S./Canada irrespective of local partner
- Academic groups^a^
- Non-profit groups^a^
- Faith-based groups^a^
- Other groups^a^

^a^group represented by at least one stakeholder at the inaugural meeting in March 2015

^b^contact established, but did not attend March 2015

Table 2 summarizes the overall timeline following these decisions. In addition to monthly steering committee teleconferences starting from July 2014, several in-person visits by different members of the group were instrumental in advancing coalition development. August 2014 saw two different STEGH partners (one academic institution, one non-profit organization) visit La Romana together to discuss the subject to with local partners and other potential key local stakeholders for invitation. A number of local leaders and professionals were also recruited to play an on-the-ground role in promoting the initiative, and community feedback was recorded in a debrief report circulated to steering committee members.

**Table 2:** Timeline of development towards COSACO

Sept 2011
First meeting with key U.S. partners at the mission-hospital level
Early 2012
Monthly teleconferences and e-mail list-serve for information exchange begin
Oct 2012
First symposium held at academic institution in New England with three partner organizations.
Oct 2013
Second symposium held; improved attendance. Mission hospital invited, but unable to attend.
Feb 2014
Third symposium held with nearly 1/3^rd^ of STEGH teams plus local mission hospital partners there. Discussions highlight need for greater information sharing.
May 2014
Attendees from the symposium are at a major global health conference; during presentations, identify other attendees not part of the symposium collaboration that still work in La Romana; exchange contact information and agree to discuss ways to work together
Jul 2014
Initial teleconference held amongst newly expanded group. Suggestion to develop a local healthy community partnership to improve STEGH work.
Aug 2014
Two partners visit La Romana and meet with key stakeholders (local government, public health, non-profit stakeholders, university partners) to pitch HCP idea and obtain feedback and buy-in.
Sep 2014
A different partner from the newly expanded group visits La Romana and obtains buy-in from their local partner while reinforcing messaging to partners already contacted. Debrief teleconference held among expanded group agrees to two targets: symposium in November 2015 and pre-meeting March 2015
Oct 2014 – Feb 2015
Participant list developed; invitations extended; pre-meeting planned and final last minute prep takes place over an alternative spring break in February 2015.
March 2015
Inaugural meeting of COSACO.

In September, another visit by an academic partner on the steering committee obtained additional buy-in from community leaders. At this time, the decision was also made to proceed with a planned meeting, tentatively set for March 2015. In keeping with local community practices the steering committee agreed that the language employed in coalition meetings would be Spanish.

Bilingual invitations to the pre-meeting were subsequently issued to partners in January 2015, and a final visit by one of the steering committee members allowed the provision of last minute invites in person, with many key face-to-face meetings (e.g. with the head of the local government) undertaken to emphasize their importance to the process.

### Birth of COSACO: first meeting

March 2015 saw a 2-day meeting in La Romana attended by representatives of the steering committee institutions and other invited participants from the local community (as indicated in Table 1). Facilitated by a bilingual representative from one of the visiting academic institutions, the group participated in a combination of forum and small group discussions.

Initial discussions laid out the concept and form of an HCP in La Romana, with subsequent small group work focused on developing options for a name, vision, and goals, all of which were subsequently voted on by the wider group. The selected goals (Table 3) reflected the composition of stakeholders present and the opening discussion, and were evenly divided between HCP development and next steps (e.g. inviting other key players), and the actual work of the HCP (e.g. educating and involving *batey* citizens about community health).

**Table 3:** Initial goals set out by the COSACO steering committee at the inaugural meeting

1. Create a database of institutions that work in communities including work statistics
2. Involve the community in all aspects of collaboration in a participatory manner and respect their autonomy and culture
3. Educate and empower the community to be self-sustaining
4. Define a set of indicators to measure the success and impact of collaboration
5. Increase awareness of the work of COSACO among health authorities, organized groups and the community at large and solicit their support and collaboration.

Small group work then aimed to develop rough work plans towards achieving these goals, which were then endorsed by the larger group. At the close of the meeting, partners agreed to formalize the steering committee, structured with one representative from each organization and a chair selected by consensus. The chair ultimately selected was the incumbent from the steering committee that planned the meeting, and the steering committee was tasked with overseeing the work of various working committees that would achieve the identified goals ahead of the next meeting. Two follow-up meetings were identified as milestones: a June meeting between the local partners alone, and another annual meeting of the wider group to be held in spring 2016. With that, a coalition for community health was officially forged.

## Results

The application of a modified HCP framework in La Romana subjectively provided several benefits, some immediately realized, and others with future potential. Initial planning discussions between STEGH-conducting institutions quickly revealed a similarity of activities: shared in common were local partners, locales of work, and shared areas of interest applied to various research and projects.

One immediate benefit was the establishment of a community of practice that otherwise did not exist, allowing stakeholders to understand that their work did not occur in a vacuum, fostering information exchange, and providing a forum for ideas, all while allowing constituent groups to explore potential new collaborative strategies to achieving community health goals.

Value was also observed in the initial consultations with local partners; the repeated invitations and check-ins highlighted a desire on the part of visiting institutions to shift the paradigm of STEGH in the community towards privileging local input. Many community partners quickly understood the desire to focus on the root causes of ill-health instead of providing temporary assistance and appreciated the push for greater community leadership, guidance, and oversight of coordinated STEGH.

COSACO’s first meeting thus stood as a sentinel example of the power of communication arising from the formation of a partnership. Informal evaluation following the meeting suggested that simply bringing the charter stakeholders into discussions allowed sharing of information about each other’s activities, identification of jointly-held opportunities, and examination of more lasting solutions to existing health and health systems issues in the *bateyes*. Interestingly, many of the local stakeholders that had been working long-term with STEGH groups discovered that a long-term external focus had often prevented them from investing into relationships with other community groups in the coalition that worked on similar issues. Many groups expressed initial concerns that a direct import and application of the HCP model would not be successful, notably concerning competition between local stakeholders for scarce resources and also the conflicting motives of stakeholders, particularly those that did not attend the initial meeting. However, these partners were more than happy to offer advice on how to better adapt the model to the local Dominican context.

In addressing specific concerns around the conduct of STEGH, the discussion provided value in securing agreement among all concerned that the conduct of unsustainable, service-focused STEGH was not in the best interests of the community. Like other HCPs initiated elsewhere, stakeholders easily understood and agreed with the concept that “health is more than just health care”, though many acknowledged that addressing root causes of health problems in the *bateyes* would be difficult. It was also noted that the existence of the HCP had the potential to provided scale to reduce barriers and create solutions, be it around a critical mass of partners for broader, more systems-level changes, or in adding voices together in advocating as a coalition.

In evaluating the process during the steering committee de-brief, four critical factors were identified as having contributed to the successful recruitment and involvement of stakeholder representatives in this initial HCP meeting in a STEGH setting:

1. A diverse, well-connected steering committee that employed its broad network to influence other stakeholders to participate, led by individuals with complementary skills (e.g. skills in bilingual facilitation abilities; consensus/negotiation; community knowledge and expertise, etc.).
2. Repeated invitations and follow-up from the initial steering committee to a broad group of stakeholders with some coherence of values, vision, and programming for the community in question.
3. Easy to understand issues (uncoordinated STEGH and a desire to improve the health of the local community) and engaged local community stakeholders to assist with framework adaptation.
4. Willingness on the part of all stakeholders to collaborate and consider their work as part of a greater whole; important for broad discussions on a shared vision and purpose.

The next step will be for COSACO to build on the identified goals from the preliminary meeting and recruit other key local stakeholders, such as the local government, health authorities, and the private sector (specifically, the largest sugar cane company, which employs the workers in the *bateyes*). This will help to broaden discussions by allowing these stakeholders to contribute to a shared vision for health in the *batey* community. Of note, the local governor had been invited by one of the visiting STEGH groups a month ahead of the meeting and had agreed to come, but was unable to attend in the end due to a conflict in his schedule.

Regardless of outcome, the very existence of COSACO has the potential to disrupt the current means by which STEGH occur in La Romana. COSACO now serves as a forum for visiting organizations on STEGH to obtain broad community guidance on a number of initiatives. Local organizations that previously targeted their community work to a select number of visiting STEGH teams can now work together towards a common goal. The potential is immense, and continued conversation will undoubtedly result in greater collaboration, opportunities for joint advocacy, and greater integration of the work of STEGH teams into established community health and development projects in La Romana.

## Conclusions

There is enormous potential for a properly applied modified Healthy Community Partnership framework to integrate visiting STEGH that would otherwise exist outside the formal health system of host communities. These partnerships have the potential to break down silos within both visiting and local stakeholder groups, enable information exchange, and provide locally relevant guidance and direction in integrating STEGH into broader community health plans.
